# Accumulation and Inspissation of Cerumen

**Published:** 1875-10

**Authors:** Swan M. Burnett

**Affiliations:** Knoxville, Tenn.


### ACCUMULATION AND INSPISSATION OF CERUMEN.

BY SWAN M. BURNETT, M. D., KNOXVILLE, TENN.

*Read Before the Knox County Medical Society.*

Cases of inspissation and long retention of cerumen in the external auditory canal are not rare. But the very fact of their common occurrence should give them an interest to the general practitioner. It is well for him to be able to diagnose cases of accumulation of wax from other affections of the ear, for often the symptoms so much resemble those given by other aurol troubles—and some of them of a very serious nature—that it is frequently highly important to decide with positiveness whether the symptoms arise *solely* from the accumulation of cerumen, or whether there is some more serious affection complicating it.

A case that occurred recently in my practice is an instance in point:

Esqr. J., æt. 48, has had trouble with his left ear for several weeks. Complains of noises and a stuffed feeling in the ears; is at times better, and the ear has on several occasions "opened" with a crack when he coughed or sneezed, and after this the symptoms were relieved to a greater or less extent for sometime. There is no pain. The watch, in that ear, can only be heard at 3 inches. The right ear about normal. The tuning-fork placed on the forehead was heard loudest in the affected ear. There was some wax in the internal auditory canal, hiding the membrana tympani. I ordered him to instill warm water into it frequently, and to return next day.

On his return the following day, I found that by performing Valsalra's experiment, his hearing-power was increased slightly, and that the noises were somewhat diminished.

I now proceeded to remove the wax by syringing, and obtained a large quantity, a piece of considerable size coming away after throwing the water in with some force. Immediately after it escaped, he said he felt quite dizzy and was nauseated. These symptoms of vertigo and nausea, I would here remark, are quite frequent accompaniments of an increase, and particularly a sudden increase, of intra-auricular pressure. In this case it resulted from the sudden pressure of the stream of water from the syringe upon the membrana tympani, which was transmitted by the chain of bones to the labyrinthine fluid. This symptom, however, passed away in a few seconds, and he remarked that the noises were all gone, and that his hearing-power was perfectly restored, which, upon applying the test, I found to be the case. On inspection, I found the membrana tympani of its normal shape, the light spot of normal size and position, and the color natural, with the exception of a vessel running down the handle of the malleus. Since that time he has had no further trouble.

Here was a case where the accumulation of wax had gone on unnoticed for years, it may be, and it was only by the supervention of a slight catarrh, of the tube causing its closure, and thus reducing his hearing acuteness still lower, that his attention was called to his ears. In fact, the symptoms of tubal catarrh were so prominent as to mislead me for the time, and caused me to refer all the trouble to that cause. As the removal of the cerumen completely relieved him, I was compelled to consider the tubal catarrh as a secondary affair.

A common popular error, and one that but too often finds lodgment in the mind of the general practitioner, is to refer almost all the ear troubles to the accumulation of wax in the external auditory canal. I need not warn the gentlemen of this society against falling into any such error. They are too accustomed to looking at the connection of cause and effect to embrace any such absurd doctrine as that. In my experience, accumulation of cerumen is far less frequent than many other affections of the auditory apparatus. Take the reports of hospitals and institutes where ear affections are treated, and you will find that catarrhal troubles of the middle ear and tube, and their complications and results, constitute by far the largest number of cases. Nevertheless, accumulation of wax does often occur, and it is highly important that we should know whether that is the *sole* trouble, and that we be able to treat it successfully.

As a basis of what few observations I propose to make before this society this evening, I will relate a most interesting case that is at present under my care.

Mrs. Jane H., about 30, has not heard well out of her left ear for many years—for twenty, she thinks. It has never, until recently, given her any pain, but for a long time she has been troubled with *tinnitus aurium*. Some two months ago, she applied to me for treatment, and on throwing the light from the mirror into the ear, I found the canal almost completely filled with cerumen. I endeavored to remove it by syringing, but with no success—not a particle could I get away. I then sent her home with instructions to instill warm water frequently, and report next day. I still found no softening of the mass. I then applied glycerin, but with a like want of success. Her ears then began to give her some pain, and I attempted to remove the mass with a small cataract spoon, but found the parts so tender that I was compelled to desist. There was one spot, very small, on the lower wall of the canal that was extremely tender. The mass was very tough—almost like India-rubber. I attempted to break it up by means of a cataract needle, but was unsuccessful in that likewise. I prescribed an alkali solution to be used, but the pain after this last examination was so great that it was not used. As a consequence of the swelling of the mass from imbibition of the liquids poured into the ear, and it may be from the instrumental interference (though they were used with great care) a very severe external attites set up, and for weeks the intense pain gave her scarcely any rest. Her nights were sleepless and her days were, for the most part, full of agony. The external auditory canal was completely closed by the swelling of its walls, and no attempt could be made to remove the mass. The only thing that could be done was to palliate, and this was attempted by means of hot applications to the ear, the instillation of hot water into the external auditory canal, and opiates. But these gave only a moderate relief. Suppuration soon set in, with a free discharge of pus, which was followed by a mitigation of the symptoms. This was kept up for weeks, the pain sometimes very severe and at others almost nil. The parts were exceedingly tender, so as to make any manipulation entirely out of the question—even syringing with moderate force was not bearable. This tenderness was still most prominent at the point on the lower wall of the meatus above referred to. After the discharge of pus had continued for some two weeks, there was noticed a whitish prominence of a conical shape springing from this point. Upon touching it with a needle, it bled rather profusely. It was a polypoid growth arising from a granulating surface beneath. In a few days thereafter, while washing her ear, she removed a small scale of bone, and the next day two more, which she brought to me. They were about one-fourth of an inch long, and one-eighth of an inch wide. About this time she also removed masses which she supposed were "rotten flesh." As I did not get an opportunity to examine these with the microscope, I am unable to say whether she was right in her surmise, or whether they were masses of cerumen.

From this time the symptoms gradually abated—the pain became less and the tenderness of the parts very much diminished. In her syringings she brought away several masses of a whitish gluten appearance—which did not look like wax, she said—one of which she brought to me. I placed a portion of it under the microscope and found it composed largely of epithelial cells and cholesterin. I examined the ear and saw a large mass of the same gluten substance lying in the canal, which I removed. In the center you see what is readily recognized as cerumen, but completely surrounding it is this white, pearly substance, which, under the microscope, shows most beautiful cholesterin crystals.

In none of the authors that I have been able to consult, is the presence of cholesterin in the cerumen mentioned, except by Trottsch. He observes that "the surface of olden plugs is sometimes as brilliant as mother-of-pearl, from the cholesterin existing between the layers of epidermis," (p. 81.) The cause of its presence here as well, indeed, as in the brain, spinal cord, bile, etc., is still a question which pathologists and physiologists have failed to settle satisfactorily for us. Since the removal of the mass, she has not suffered any more pain, though the hearing has not improved much, being just able to hear the watch in contact with the auricle, as has been the case since the inflammation first set up. I should have mentioned that the tuning fork placed on the forehead was heard best in the affected ear. There is evidently more cerumen yet in the canal, for the membrana tympani is not yet visible, and the noises still continue. These I hope to get away in a few days, and when the canal is cleared I expect to find the membrana tympani affected, perhaps perforated.

This case is worthy of attention on several accounts. 1. The length of time the cerumen has been accumulating; 2. The results in ulceration of the walls of the meatus and exfoliation of bone; and 3. In the presence of such a large amount of cholesterin, which would be likely to mislead one who was not on the look-out for it, into supposing that it was some other morbid product.

Generally the most marked symptom, subjectively, of accumulation of cerumen, is hardness of hearing, generally coming on gradually. To this is added often, *tinnitus aurium*, or noises in the ears. The cause of those symptoms is mechanical, and of course are patent to every reflecting mind. The wax being a bad or a non-conductor of sound, cannot transmit the ærial undulations to the membrana tympani, and, consequently, when it is large in amount, imposes a complete barrier to their passage by way of the external meatus, and the only vibrations that can reach the expansion of the nerve in the labyrinth must be those that are conveyed through the bones of the skull. The tinnitus is the result always of a pressure on the terminal expansion of the nerve, which, in this case, is produced by the pressure of the accumulated wax upon the membrana tympani, which is conveyed in the manner before described to the fluid in the labyrinth, causing an increase of intra-auricular pressure. This being the case, the perceptive apparatus not being the one at fault, (except as a rare complication,) we have only to remove the obstruction to give our patient complete relief, not only to his ear symptoms, but often to his anxiety of mind, for frequently these patients labor under the fear that the nerve is affected, and the more so as a marked deafness often follows some loud sounds, or something to which their fancy can refer the origin of the trouble. The case of an artillery officer whom I relieved some months ago, is an instance in point.

Some two years ago he was engaged in practicing with his guns on the Pacific coast, and was doing some heavy firing. He stood to the left of the gun, and always turned his left side to gun to watch the effect of the shot. From this time he dates the beginning of his trouble, which was simply a difficulty in hearing in that ear. There was no tinnitus. He was fearful that the heavy firing had permanently injured the nervous apparatus. I placed the tuning fork on the forehead, and as he remarked that it sounded loudest in the affected ear. I relieved him at once of his fear by telling him that the nerve was unaffected. On inspection of the meatus, I saw at once the cause of his trouble to be accumulation of wax. This, after considerable difficulty, was removed by syringing, and his hearing power, which before was limited to the watch on contact, rose to the watch at three feet. He has had no trouble since.

In my report of these cases you will have observed that I made use of the tuning-fork placed on the forehead. This is one of the most valuable means we have of testing the integrity of the auditory nerve, and demonstrating an obstruction to the passage inwards of the ærial undulation *per riam naturolam.* If the external auditory meatus be closed by the finger, and the tuning fork placed on the forehead in a person of normal hearing power, the sound will be heard loudest in that ear. The same is true if the meatus is filled with wax, or if the middle ear is occupied with some morbid product. The cause of this phenomenon is still *sub judice.* Several explanations have been offered—the most plausible one being that of Politzer. According to his view it is most probably caused by an increased resonance, the result of the closure of the meatus, and an impediment to the egress of the sound nerves. Consequently, when the fork or a watch is placed on the forehead or teeth, and the sound is heard loudest in the ear affected, we can at once give assurance that the nerve is intact, and that the trouble lies in an obstruction in the conducting apparatus.

This expedient is often of use in the detection of simulated deafness, and it will be well for our examining pension-surgeons to bear this in mind. If the patient is a maligner, and says both ears are affected, cause him to close one ear with the finger, and place the tuning-fork on the forehead, and, as he would naturally suppose that the hearing in that ear would become worse by so doing, he will say so; whereas, in truth, it will be either not made worse if really affected, or if not affected, will be increased. If only one-sided deafness is complained of, the simulation is still easier detected. Suppose the left is the ear complained of, the right being normal. On examination we find no collection of wax, and no evidence of inflammation in the middle ear. Close the healthy ear, and if he persists that the tuning-fork is not heard at all, he is, without doubt, a maligner, for as we have seen the sound of the fork should be increased by closing the healthy ear.

I will conclude these hasty remarks by a few words upon the treatment of inspissated cerumen. The object, of course, is the removal of the mass. But simple as it may, at first sight, appear to be, it is not an easy matter sometimes to do, as witness our last case but one. The safest method, and the one that should always first be tried, is syringing. A simple enough procedure, you say, but like everything else in the world, to be effectual, it must be done in the proper manner. A few directions as to its proper performance would, perhaps, not be out of place here. If the mass looks hard, it is always well to soften it by means of warm water, instilled frequently in the ear, or what is better, an alkaline solution, as of soda and potash. After doing this for twelve to twenty-four hours, you can begin the syringing, and in the following manner: take the upper and posterior portion of the pinna between the thumb and forefinger of the left hand, and draw it upward and backward.

By this you draw the external meatus in the same direction and thus straighten the canal. The stream from the syringe is thereby thrown more directly upon the mass, and the exit of the water, and the cerumen with it, is greatly facilitated. If this simple procedure is neglected, much of love's labor will be lost. The stream should be thrown in with considerable force, and repeated until the whole mass is removed. If this is not successful, as a dernier resort you may use instrumental means, of which the scoup is perhaps the best, but in its use too much care and gentleness cannot be observed, for even then, as we have seen in one of our cases, the use of instruments is liable to bring on a severe external attitis.

